# Conference Reports: TWELFTH NATIONAL COMPUTER SECURITY CONFERENCE Baltimore, MD October 10–13, 1989

**DOI:** 10.6028/jres.095.023

**Published:** 1990

**Authors:** Elizabeth B. Lennon

**Affiliations:** National Computer Systems Laboratory, National Institute of Standards and Technology, Gaithersburg, MD 20899

The National Computer Systems Laboratory (NCSL) of the National Institute of Standards and Technology (NIST) and the National Computer Security Center (NCSC) of the Department of Defense (DoD) co-sponsored the Twelfth National Computer Security Conference held in Baltimore, Maryland on October 10–13, 1989. The theme of the conference, “Information Systems Security: Solutions for Today—Concepts for Tomorrow,” highlighted the broader focus of information systems security which now challenges the user community, vendors, system developers, and administrators. Major areas addressed included advanced research developments and emerging technologies, network security architectures, risk management, management and administration issues in computer security, and an expanded focus on education and ethics. More than 2,000 attendees from government, industry, and academia participated in the 4-day conference, which was co-chaired by NCSL’s Irene Gilbert and NCSC’s George Mitchell.

## 1. History of the Conference

The National Computer Systems Laboratory has played a vital role in protecting the security and integrity of information in government computer systems through its Computer Security Program since the passage of the Brooks Act in 1965. Since 1972, the Laboratory has issued standards and guidelines on the cost-effective protection of unclassified information in computer systems. NCSL works with organizations from government, industry, academia, and voluntary standards groups to develop standards and guidance, produce tests to evaluate conformance to standards, transfer technology to users, and provide technical assistance to government and industry in computer security applications. The Computer Security Act of 1987 strengthened NCSL’s role in protecting unclassified sensitive information in federal computer systems.

The Department of Defense also has pursued an active computer security program over many years. In 1978, the Assistant Secretary of Defense for Communications, Command, Control and Intelligence established a Computer Security Initiative to ensure the widespread availability of trusted ADP systems within the DoD community. In 1981, the National Computer Security Center was created to administer the activities of the initiative. NCSC advances the development of trusted computer systems and publishes guidelines on computer security. In response to the increased emphasis on computer security in recent years, NCSC expanded its efforts to support basic research for the development of additional trusted systems. NCSC’s technology transfer program ensures that other federal agencies and industry benefit from technological advances in computer security.

Sharing common goals and mutual challenges, NCSL and NCSC joined forces in 1979 to co-sponsor the first National Computer Security Conference. Dr. Dennis Branstad, NCSL (now a NIST Fellow) and Mr. Stephen Walker, then Chairman, Computer Security Technical Consortium, organized the first seminar as an information exchange on computer security issues. Since its inception, the meeting has expanded significantly in size and scope to become a large, comprehensive computer security conference. This development parallels an increased national awareness of the need for computer systems security and an expanded interest in existing and emerging technologies available to protect vital information resources.

## 2. The Twelfth National Computer Security Conference

The Twelfth National Computer Security Conference was organized into four tracks: research and development; systems; management and administration; and education and ethics. Of particular interest was the expanded focus on computer security education and awareness resulting from requirements of the Computer Security Act of 1987 and the in-depth concentration on computer security ethics in the workplace. The first-day “Overture” sessions offered an introduction to basic computer security subjects including an overview of agency security plans submitted in response to the Computer Security Act, ethical conflicts in computer science, NCSL/National Security Agency (NSA) joint efforts, the Secure Data Network System, DoD Trusted Computer System Evaluation Criteria, and training guidelines.

The second day of the conference opened with welcoming remarks from NCSC Director Patrick R. Gallagher and NCSL Director James H. Burrows. Representative Tim Valentine of North Carolina presented the keynote address on the role of Congress in computer security. Valentine cited the progress of federal agencies in implementing the Computer Security Act and called for expediting federal progress in computer security technology and standards, including international standards. An opening plenary on “Information Systems Security—A Year in Review,” followed. Participants included James Burrows, NCSL; Patrick Gallagher, NCSC; Clive Blatchford, ICL, United Kingdom; Steve Kent, Bolt, Baranek, and Newman; Stephen Walker, Trusted Information Systems; Stuart Katzke, NCSL; Eliot Sohmer, NCSC; and Harold Segal, Office of Personnel Management. Among the subjects addressed were legislation and policy; the international standardization effort; electronic data interchange; trusted systems, virus response centers; international trusted criteria; and computer security training and awareness.

Following this overview, speakers from government and industry presented concurrent sessions in the four tracks described above. Selected presentations are summarized below.

### 2.1 Track A: Research and Development

#### 2.1.1 Database Management

Achieving multilevel database security was the focus of the database management session chaired by Teresa F. Lunt of SRI International. Gary W. Smith of George Mason University presented a paper describing a balanced approach which uses good system design, management controls, and procedural security as well as technical solutions to achieve multilevel database security.

Tim Wood of Sybase, Inc. gave an overview of the system architecture of the Sybase Trusted SQL Server, targeted at the B2 level of trust. The Trusted SQL Server is a physical machine control program that is a hybrid of a secure, high-performance DBMS server with a dedicated kernel of original design. A third paper by R. Alan Whitehurst, University of Illinois at Urbana-Champaign, and Teresa F. Lunt, SRI International, discussed the verification of the SeaView formal top-level specifications and the benefits gained from formally specifying and verifying selected database operations. The SeaView project was a 3-year program to create the design of a multilevel secure relational database system that met the criteria for trusted system Class Al.

#### 2.1.2 Verification Methodology

Joshua Guttman, MITRE Corporation, chaired this session. Carla Marceau and C. Douglas Harper, Odyssey Research Associates, presented a paper describing an interactive approach to Ada verification. Penelope is a prototype Ada verification editor whose user interactively and concurrently develops specifications of programs, their Ada text, and proofs of their verification conditions. Adding CASE technologies to formal verification was the subject of the next paper by J. V. A. Janeri, J. S. Barlas, and L. L. Chang of the MITRE Corporation. The authors used CASE technology to further automate the labor-intensive task of formal verification, by integrating the process of formal software design verification with the software engineering life cycle. The result is a Specification Browser which serves as a verification aid. In the final paper of this series, Timothy E. Levin, Steven J. Padilla, and Roger R. Schell, Gemini Computers, Inc., presented engineering results from the trusted system level Al formal verification process. The authors reviewed the formal methodology used to verify the security of the GEMSOS TCB, currently under development and targeted for the TCSEC Class Al level.

#### 2.1.3 Verification

Marvin Schaefer, Trusted Information Systems, chaired the verification session. Barbara A. Mayer and Monica M. McGill, NCSC, presented an overview and rationale of recently published guidelines for formal verification systems. The paper described the history and status of the guidelines, the endorsement process, the evaluation approach, and possible future directions for verification systems.

A second paper by William D. Young, Computational Logic, Inc., compared and contrasted the Gypsy and Z specification languages. The authors suggested refinements to the two languages and pointed a direction for future language designs. The final paper in this group evaluated security model rule bases; John Page, Jody Heaney, Marc Adkins, and Gary Dolsen, Planning Research Corporation, are the authors of the paper. The evaluation viewed three different security models from the common point of reference provided by the Security Model Development Environment prototype.

#### 2.1.4 Models

The session on models was chaired by D. Elliot Bell, Trusted Information Systems. In the first presentation, Robert S. Lubarsky, Franklin and Marshall College, described a mathematical approach to hook-up security and generalized restrictiveness. A second presentation explained the Argus computer security model.

#### 2.1.5 Security Architecture

NCSL’s Lisa Carnahan and NCSC’s Mario Tinto chaired these sessions. Papers focused on a broad range of products and systems: the design of trusted workstations using a total “information security” (INFOSEC) solution; Formal Top Level Specification (FTLS) security testing for the Honeywell LOCK project; the formal specification of security aspects of a messaging system architecture; a Secure Distributed Operating System (SDOS) prototype; a high B level security architecture for the IBM ES/3090 Processor Resource Systems Manager; and a TRW security engineering effort to define an architecture for a MLS communications processor. An R&D panel and an ethics plenary session concluded the track.

### 2.2 Track B: Systems

Network security architectures received in-depth coverage, focusing on privacy and access control issues. Recent incidents involving malicious code have drawn increased attention to the need for prevention and remedies in this area.

#### 2.2.1 EMail and Authentication

Ruth Nelson, GTE, chaired this session. John Linn, DEC, and Stephen Kent, BBN Communications Corporation, presented a paper on privacy for DARPA-Internet mail. The facilities discussed provide privacy enhancements on an end-to-end basis between originator and recipient User Agent processes, which may be implemented on heterogeneous systems. The authors defined and recommended a cryptographic key management approach employing RSA-based public-key certificates.

Key management and access control for an electronic mail system was the subject of the next paper by Martha Branstad, W. Curtis Barker, Pamela Cochrane, and David Balenson of Trusted Information Systems, Inc. The authors examined key management and access control services associated with the Embedded Network Security (ENS) Trusted Mail system, indicating how both encryption and trusted system functionality provide protection. Miles Smid, James Dray, and Robert Warnar, NCSL, described a token-based access control system for computer networks. In this system, a user’s access is mediated by a smart token implementing a transparent cryptographic three-way handshake with the target computer.

#### 2.2.2 Local Area Networks

The increasing use of local area networks (LANs) has driven the search for cost-effective security solutions. Dennis Branstad, NCSL, chaired the session on LANs. Gary Stoneburner and Dean Snow, Boeing Aerospace and Electronics, described how and why the Boeing Multilevel Secure local area network (MLS LAN) is migrating towards an Information Security (INFOSEC) solution. Significant design issues were presented, as well as an overview of how encryption might be embedded into the MLS LAN. L. Kirk Barker, Datotek, and Kimberly Kirkpatrick, MITRE, next described the Standard for Interoperable LAN Security (SILS) model which would provide a standard protocol for protecting LAN traffic; the IEEE 802.10 is basing its security protocols and services for LANs on this model. Peter Loscocco, NCSC, presented the last paper in this series on a dynamic network labeling scheme for a MLS LAN.

#### 2.2.3 Networks

Donna Dodson, NCSL, chaired the networks session addressing access control; Dennis Grayson, NCSC, chaired the second networks session. Extending mandatory access controls to a networked multilevel secure (MLS) environment was the subject of the first presentation by Ron Arbo, Eric Johnson, and Ron Sharp, AT&T Bell Laboratories. They introduced a software package design that permits MLS systems to securely communicate without modifying or trusting the existing network applications. Richard Graubart, MITRE, reexamined the traditional access control policies and proposed a new type of access control policy. Four DEC researchers next described a digital distributed system security architecture. Other papers in this series outlined guidelines for specifying security guards and the security of embedded tactical systems.

#### 2.2.4 Computer Viruses and Related Threats

Protecting information systems from threats of all kinds was the focus of these sessions; Jack Holleran, NCSC, and James Anderson, J. P. Anderson Co., were session chairs. Martha Brothers, AT&T, gave a “how to” guide for virus protection in MS-DOS, while Ronald Tencati, Goddard Space Flight Center, and Patricia Sisson, Science Applications Research, described the “Father Christmas Worm” of December 1988 which invaded a large DECnet network and reached 6,000 computer nodes worldwide. Cliff Stoll, Harvard—Smithsonian Center for Astrophysics, gave an epidemiology of viruses and network worms. Other talks summarized computer crime techniques and gave potential solutions.

The systems track concluded with a discussion of vendor activities by Dennis Sirbaugh, NCSC, and a plenary session on ethics.

### 2.3 Track C: Management and Administration

#### 2.3.1 Management

Irene Gilbert was session chair for management. William Norvell, Hughes Aircraft Company, gave the first presentation on integrating accreditation activities into the acquisition process, ensuring that all security requirements are specified in the functional baseline for design and test. David Juitt, Digital Equipment Corporation (DEC), proposed a security approach through system management; he detailed an ongoing advanced development effort within DEC to study security issues of computing across a worldwide distributed environment and how they relate to conducting business safely. A third paper described a systematic approach to software security evaluations.

#### 2.3.2 Accreditation

In this session chaired by Grant Wagner, NCSC, the first presentation by Toni Fish, Information Systems Security Association, and Corey Schou, Idaho State University, addressed the issue of the certification of computer security professionals. Darryl Song, MEI Associates, spoke on the accreditation of information systems and networks, followed by a talk by Horace Peele, Electronic Security Command (ESC), USAF, on the development of the ESC Accreditation Package. Peele recommended automated accreditation packages as effective security tools throughout the Department of Defense and the federal government.

#### 2.3.3 Risk Management

These sessions were led by Sylvan Pinsky, NCSC, and Irene Gilbert, NCSL. Jennie Stevens and Richard Weiner, Booz, Allen & Hamilton, Inc., presented an innovative concept for computer security risk assessment developed in 1988 and 1989 by their company. The concept provides a framework upon which an individual organization’s customized guideline can be built. Next, Suzanne Smith, Los Alamos National Laboratory, introduced LAVA, the Los Alamos Vulnerability/Risk Assessment system, a three-part systemic approach to risk management that can be used to model a variety of application systems. A third paper focused on the purpose and framework of anomaly detection; G. Liepins, Oak Ridge National Laboratory, and H. Vaccaro, Los Alamos National Laboratory, placed anomaly detection of computer use in the framework of overall computer security. NCSL’s Stuart Katzke gave an update of progress in the federal government’s risk management activities.

The remainder of Track C dealt with Air Force customer programs, a “speak out” session for the informal exchange of ideas and opinions, an outline of NCSL programs by Miles Smid, and a concluding plenary session on ethics.

### 2.4 Track D: Education and Ethics

This new track on computer security education and ethics attracted much interest.

#### 2.4.1 Ethics Overview

Charles Pfleeger, Trusted Information Systems, chaired the overview session on ethics. In the first presentation, Larry Martin, Subcommittee on Automated Information Systems Security, addressed the issue of responsibility for unethical computer behavior. Martin concluded that while the ultimate responsibility to behave in an acceptable manner belongs to the user, all of us share in the responsibility for and the consequences of unethical computer use. Glenn D. Watt, NCSC, spoke next on the ethical dilemma of malicious code; his solution combines technology and ethics. A panel discussion followed.

#### 2.4.2 Management Responsibility vs. Individual Rights

Management responsibility versus individual rights was the focus of the next session, chaired by Lance Hoffman, George Washington University. The first speaker was Robert Veeder, Office of Management and Budget, whose topic was the Computer Matching and Privacy Protection Act of 1988. Anna Patrick, U.S. Department of Agriculture, spoke next on public access to government databases. A panel discussion followed on the role of management versus the prerogatives of individuals; panel members included session speakers plus Brian Hyland, U.S. Department of Labor; Jan Goldman, ACLU; and Marc Rotenberg, Computer Professionals for Social Responsibility.

#### 2.4.3 Criminalization of Computer Abuse/Misuse

William Murray, Ernst & Whinney, chaired the next session on the criminalization of computer abuse and misuse. Jay BloomBecker, National Institute on Computer Crime Data, discussed trends in computer misuse, followed by James Miller, University of Southern Mississippi, who gave an academic perspective on computer abuse. A prosecutor’s point of view was presented by William Cook, U.S. Attorney’s Office; he successfully prosecuted the first case under the Computer Fraud and Abuse Act of 1986. A panel discussion concluded the session.

#### 2.4.4 Ethics in the Workplace

Who is responsible for ethics in the workplace? James P. Anderson, James P. Anderson Co., led the session addressing this issue. Karen Forcht, James Madison University, presented a talk on the ethical use of computers, followed by a panel discussion.

#### 2.4.5 Education, Training and Awareness

At this point, the focus of Track D shifted to education, training, and awareness issues in computer security. Lauresa Stillwell, Department of State, chaired the session. W. V. Maconachy, NSA, discussed turning a philosophical orientation of computer security education into a practical reality. He challenged government personnel to develop and implement a well-orchestrated government-wide information systems security awareness, training, and education model. A panel then compared and contrasted the education, training, and awareness continuum, followed by a talk by John Higgins, Brigham Young University, on training computer science undergraduates in information security.

Harold Segal, Office of Personnel Management (OPM), led the session on computer security training in the federal government. Following his talk on OPM training modules, Anne Todd, NCSL, presented the training guidelines which she coauthored. A federal training panel concluded the session.

The computer security awareness session was chaired by Harry DeMaio, Deloitte, Haskins & Sells. His talk on employee awareness was followed by one on executive awareness by Joan Foreman, Bureau of Engraving and Printing. An executive awareness panel preceded the closing talk on mandated versus voluntary ethics given by Marlene Campbell, Murray State University (Kentucky).

## 3. Summary of the Conference

The Twelfth National Computer Security Conference covered a broad range of issues and emerging technologies of value to those charged with the responsibility of protecting vital information resources in computer systems. “Information Systems Security: Solutions for Today—Concepts for Tomorrow” offered guidance for the present and planning strategies for the future. In the rapidly changing world of information systems technology, the importance of planning for the security of tomorrow’s information systems is critical to all who understand that an organization’s information is its most valuable asset.

Conference proceedings are available from conference co-chair Irene Gilbert, National Computer Systems Laboratory, A216 Technology Building, National Institute of Standards and Technology, Gaithersburg, MD 20899, or you may call (301) 975-3360.

